# Human BMP4 mRNA Encapsulated in Lipid Nanoparticle for Bone and Articular Cartilage Repair in Aged Mice

**DOI:** 10.3390/jfb17060273

**Published:** 2026-06-01

**Authors:** Xueqin Gao, Zuokui Xiao, Matthieu Huard, Keisuke Nakayama, Aryn Cummings, Britney S. Force, Hongye Li, Chiara Mancino, John P. Cooke, Francesca Taraballi, Marc J. Philippon, Johnny Huard

**Affiliations:** 1 Linda and Mitch Hart Center for Regenerative and Personalized Medicine, Steadman Philippon Research Institute, Vail, CO 81657, USA; zxiao@sprivail.org (Z.X.); mhuard@sprivail.org (M.H.); knakayama@sprivail.org (K.N.); bforce@sprivail.org (B.S.F.);; 2 Laboratory Animal Resources, Colorado State University, Fort Collins, CO 80523, USA; aryn.cummings@colostate.edu; 3 Center for Musculoskeletal Regeneration, Houston Methodist Academic Institute, Houston, TX 77030, USA; hli3@houstonmethodist.org (H.L.); cmancino@houstonmethodist.org (C.M.); jpcooke@houstonmethodist.org (J.P.C.); ftaraballi2@houstonmethodist.org (F.T.)

**Keywords:** human BMP4 chemically modified mRNA, critical calvarial bone defect, osteoarthritis, age-related osteoarthritis, lipid nanoparticle, bone tissue engineering

## Abstract

Segmental bone defects and age-related osteoarthritis (OA) are clinically challenging in terms of treatment. Although preclinical studies have demonstrated efficacy for bone defect healing and OA using ex vivo gene therapy or biomaterial sustained-release delivery, few such treatments have translated into clinical therapies due to safety concerns. Bone morphogenetic proteins belong to the transforming growth factor β (TGFβ) superfamily and are effective in bone and cartilage regeneration/repair. Among BMPs, BMP4 is not only effective in promoting bone and cartilage repair but also promotes stem cell renewal potential and exhibits anti-aging effects. Therefore, the aim of this study is to investigate whether human BMP4 mRNA encapsulated in lipid nanoparticles (hBMP4 mRNA/LNP) can promote bone and cartilage repair. In vitro data demonstrated that hBMP4 mRNA/LNP-treated human MSCs secreted BMP4 protein, as detected by ELISA, and enhanced osteogenic differentiation. In vivo results demonstrated that hBMP4 mRNA/LNP at a 50 µg dose promoted limited new bone formation only at 2 weeks after creation of defect in critical-sized calvarial bone defects in aged mice when delivered using fibrin sealant hydrogel, as revealed by micro-CT and histology. However, intra-articular injection (IA) of lower doses (2.5 and 5 µg) in aged mice knee joints prevented cartilage loss, as demonstrated by micro-CT; decreased OARSI histology scores; and improved cartilage-specific matrix COL2. hBMP4 mRNA/LNP at a 5 μg dose significantly increased SOX9^+^ cells per normalized cartilage area as well as the percentage of SOX9^+^ cells in the cartilage area. hBMP4 mRNA/LNP treatment showed a trend of pain alleviation and did not change serum hyaluronic acid levels. In conclusion, human BMP4 mRNA encapsulated in lipid nanoparticles improved cartilage repair and delayed cartilage degeneration in aged mice, while having a limited effect on bone healing, even at a higher dosage. These results suggest that hBMP4 mRNA encapsulated with lipid nanoparticles represents a promising treatment for age-related OA.

## 1. Introduction

Age-related osteoarthritis (OA) affects tens of millions of patients, often causing disability and pain and affecting quality of life. Currently, there is no disease-modifying treatment. At the end stage of OA, patients often need joint replacement [1,2]. Segmental bone defects caused by trauma and tumor resection are challenging orthopaedic conditions to treat [3,4]. Autografts, allografts, and BMP2 protein therapy remain the current available clinical treatments. However, complications such as donor site morbidity, infection, and heterotopic bone formation have hindered their applications [5]. Hence, the development of novel therapies to treat osteoarthritis or segmental defects is critically needed.

To date, 20 BMPs have been identified, each with diverse and distinct roles. Previous studies have demonstrated that BMP2,4,6,7,9 could promote bone regeneration in different animal models when delivered via gene therapy or sustained-release biomaterials. BMP3 exerted an inhibitory effect on bone formation induced by BMPs 2, 6, and 7, but not BMP9 [6,7,8]. BMP2,4,6,7,9 have also been shown to promote chondrogenic differentiation of human bone marrow mesenchymal stem cells (hBMMSCs), with BMP2,4,9 being the most potent. In vivo, the sustained release of BMP2,4,6,7,9 using a Heparin/poly(ethylene arginine aspartate diglyceride) (PEAD) coacervate promoted cartilage repair in a rat microfracture-mediated osteochondral defect in rats [9]. BMP5 regulates osteoclasts, whereas a combination of BMP2,5,6 stimulated osteoblasts, not osteoclasts [10]. BMP12 [11,12] and BMP14 [13] have also been shown to promote cartilage repair.

Bone morphogenetic protein 4 (BMP4) plays diverse roles during development, including promoting stem cell renewal [14,15] and enhancing induced pluripotent stem cell (iPSC) reprogramming efficiency [16,17,18]. BMP4 has also been shown to have anti-senescent, anti-steatotic, anti-inflammatory, and anti-fibrotic properties. By contrast, its antagonist Gremlin 1, which is particularly highly expressed in human visceral fat, is pro-senescent and antagonistic to BMP4 in non-alcoholic fatty liver disease [19]. Previously, it has been shown that muscle-derived stem cells (MDSCs) retrovirally transduced to express human BMP4 improved cartilage repair in a rat model of monosodium iodoacetate (MIA)-induced OA [20]. BMP4 promoted the robust chondrogenic differentiation of human mesenchymal stem cells (MSCs) and coacervate-mediated delivery of various BMPs, including BMP4, and promoted microfracture-mediated cartilage repair in an osteochondral defect model [9]. BMP4, in combination with transforming growth factor 1 and delivered using a heparin–fibrin gel, promoted cartilage repair in a rabbit osteochondral defect model [21]. BMP4 has also reversed age-related declines in muscle-derived stem cell proliferation and enhanced bone regeneration [22]. Therefore, BMP4 is a bone morphogenetic protein that can play multiple roles in bone and cartilage regeneration. Currently, BMP4 has not been used clinically for musculoskeletal tissue regeneration and repair due to concerns associated with gene therapy-based approaches. The lipid nanoparticle-mediated delivery of chemically modified mRNA encoding therapeutic targets may offer more localized and safer targeted gene delivery over other viral therapies with less immunogenicity.

Furthermore, mRNA encapsulated in lipid nanoparticle-mediated gene delivery has become increasingly feasible following the successful development of the COVID-19 mRNA vaccines to control the pandemic. Several chemically modified mRNAs that encode therapeutic proteins have been used to repair different musculoskeletal tissues [23]. BMP2 mRNA encapsulated in lipid nanoparticles has been shown to regenerate critical-size long bone defects via endochondral bone formation, achieving outcomes equivalent to a clinical dose of BMP2 protein [24]. BMP9 mRNA complexed with nanoplex polyethylenimine (PEI) has also been shown to enhance calvarial bone defect repair and results in complete defect closure at a 50 µg dose [25]. However, most studies have been performed on young animals, which have superior tissue regeneration compared to aged animals. Hence, the goal of this study was to investigate whether human BMP4 mRNA encapsulated with lipid nanoparticles will promote bone defect healing and cartilage repair in aged mice.

## 2. Materials and Methods

### 2.1. Human BMP4 (hBMP4) mRNA Preparation and Encapsulation

In vitro transcription was performed by RNA core from Houston Methodist Academic Institute. Human BMP4 clone in pCMV6-XL4 vector was purchased from OriGENE (Cat#:SC108985) and subcloned into a plasmid containing the T7 promoter, the 5′-UTR of human β-globin (HBB), the multiple cloning site (MCS), the 3-UTR of HBB, a 151 bp poly-A sequence, and a restriction site for linearization with a type IIS restriction enzyme, following the poly-A sequence as previously reported [26] for in vitro transcription. To generate chemically modified hBMP4 mRNA, in vitro transcription used CleanCap and incorporated N1-methyl pseudo-urindine triphosphate (N1-M-pUTP) to decrease immunogenicity. The full size of transcribed hBMP4 mRNA is 1603 bp. RNA was then purified and subsequently encapsulated in lipid nanoparticles as stated below.

After hBMP4 mRNA was purified post in vitro transcription, hBMP4 mRNA was encapsulated in lipid nanoparticles (LNPs) using a clinically validated ionizable lipid formulation composed of SM-102, DSPC, cholesterol, and DMG-PEG2000 at a molar ratio of 50:10.5:38:1.5. Lipids were obtained from MedChemExpress (SM-102, Cat# HY-134541, Monmouth Junction, NJ, USA) and Avanti Polar Lipids (DSPC, Cat# 850365P; cholesterol, Cat# 700000P; DMG-PEG2000, Cat# 880151P, Alabaster, AL, USA). The aqueous phase consisted of human BMP4 mRNA dissolved in sodium citrate buffer (150 mM, pH 4.5), while the lipid components were prepared in ethanol to form the organic phase. Both phases were equilibrated at 45 °C prior to controlled mixing using a NanoAssemblr Ignite microfluidic system (Cytiva, Wilmington, DE, USA), employing a 1:3 organic-to-aqueous flow rate ratio at a total flow rate of 12 mL/min. The nitrogen-to-phosphate (N/P) ratio was maintained at 6. Following nanoparticle assembly, LNPs were dialyzed overnight at 4 °C against phosphate-buffered saline (PBS) containing 10% (*w*/*v*) sucrose, sterile-filtered through 0.22 µm filters, aliquoted, and stored at −80 °C until use. Physicochemical characterization was performed by dynamic light scattering (Malvern Zetasizer, Malvern Panalytical, Malvern, UK) to determine particle size, polydispersity index (PDI), and zeta potential. Encapsulation efficiency was assessed using a RiboGreen fluorescence assay (Invitrogen, Cat# R11490, Carlsbad, CA, USA) by comparing fluorescence signals of lysed versus non-lysed samples following incubation at 37 °C.

### 2.2. In Vitro hBMP4 mRNA/LNP Functional Test

#### 2.2.1. In Vitro hBMP4 mRNA/LNP Transfection and Osteogenic Differentiation

Human bone marrow mesenchymal stem cells (hBMMSCs) were isolated from discarded trabecular bone from an 81-year-old female who underwent arthroplasty surgery, as previously described [27,28]. HBMMSCs were provided by Dr. Rocky Tuan’s laboratory from Department of Orthopaedic Surgery, University of Pittsburgh. HBMMSCs were cultured and expanded in α-MEM supplemented with 10% fetal bovine serum (FBS) and 1 ng/mL fibroblast growth factor (FGF2) as previously described by Dr. Rocky Tuan’s laboratory [28]. hBMMSCs were seeded at 1 × 10^4^ cells/well in a 24-well plate in 4 replicates and allowed to grow to 70% confluence. Then, hBMP4 mRNA/LNP at 3 doses (1 µg/well, 2 µg/well and 4 µg/well) and freezing medium (10% *w/v* sucrose, 12 mM Tris HCl), which was used as control in serum-free medium in 0.25 mL volume, were added to each well. Cells were treated for 4 h. The supernatant was collected for measuring hBMP4 protein and stored at −80 °C until measurement. Then, 0.5 mL of normal growth medium was added to allow cell recovery. Human BMP4 protein (50 ng/mL; Cat#:314-BP/CF, R&D system, Minneapolis, MN, USA) served as a control in an additional 4 wells. At 24 h of culture, the supernatant was collected, and then 0.5 mL osteogenic medium was added as previously described [8]. Cells were cultured for another 72 h, after which the supernatant was collected. Osteogenic medium was replaced and cultured for another 2 days for a total of 5 days of osteogenic differentiation. Cells were then lysed with 1 mL Trizol for RNA isolation, down-stream cDNA synthesis, and quantitative polymerase chain reaction to detect osteogenic genes.

In another set of experiments, hBMMSCs were treated with hBMP4 mRNA/LNP at the same doses as stated above but transfected for 8 h. The supernatant was collected at 8 h, then 0.5 mL complete growth medium was added to each well, including hBMP4 protein as control. Cells were cultured for an additional 24 h, and supernatant was collected, and then osteogenic medium was added and cultured for another 72 h, supernatant was collected, and 0.5 mL osteogenic medium was added to each well and cultured for another 48 h (total of 5 days osteogenic differentiation). The cells were then fixed with 4% paraformaldehyde (PFA) for immunofluorescence staining as described below.

#### 2.2.2. RNA Isolation, cDNA Synthesis and Quantitative Polymerase Chain Reaction (Q-PCR)

The total RNA was isolated following the manufacturer’s protocol. RNA was dissolved in DNAase/RNAase-free H_2_O, and RNA concentration was measured using Tecan plate reader equipped with Nano-plate. 500 ng total RNA was used for each cDNA synthesis reaction using iScript™ Reverse Transcription Supermix (BIO-RAD, 1708841BUN, Hercules, CA, USA), and cDNA was diluted 1:2.5 using DNAase/RNAase-free water. Q-PCR was performed similarly as previously described [8] for alkaline phosphatase (ALP), osterix (OSX), runt-related transcription factor 2(RUNX2), P16^Ink4A^ (P16) and ^P21CIP/WAF^ (P21) in 10 μL reactions in duplicates using SsoAdvanced™ Universal SYBR^®^ Green Supermix (BIO-RAD, #1725272) and ABI StepOnePlus Q-PCR machine (Thermo-Scientific, Waltham, MA, USA). Human glyceraldehyde-3-phosphate dehydrogenase (GAPDH) was used as a housekeeping gene. The primers were designed with primer 3 input [29,30,31]. The primer sequences are as follows (5′−3′): GAPDH—forward: GCCTTCCGTGTCCCCACTGC, reverse: CAATGCCAGCCCCAGCGTCA, product size 211 bp; ALP—forward: AGAATCTGGTGCAGGAATGG, reverse: CATGAGATGGGTCACAGACG, product size 107 bp; OSX—forward: GCAGCTAGAAGGGAGTGGTG, reverse: AAGCCTTGCCATACACCTTG; product size 212 bp; RUNX2—forward: GGTACCAGATGGGACTGTGG, reverse: TCGTTGAACCTTGCTACTTGG; product size 111 bp; P16—forward: CTTCCTGGACACGCTGGT, reverse: GACCTTCCGCGGCATCTATG, product size 185 bp; P21—forward: CAAGCTCTACCTTCCCACGG, reverse: ATCTGTCATGCTGGTCTGCC, product size 226 bp; collagen type 1A (COL1A1)—forward: GTGCTAAAGGTGCCAATGGT, reverse: ACCAGGTTCACCGCTGTTAC. The mRNA expression levels were expressed as fold change using the CTL as the control (2^−ΔΔCT^).

#### 2.2.3. Immunofluorescence Staining

To detect cell proliferation marker, marker of proliferation Kiel 67 (Ki67), and osteogenic differentiation marker collagen type I protein (COL1) after 5 days osteogenic differentiation, cells were fixed with 4% paraformaldehyde (PFA), then washed 3 times with phosphate-buffered saline (PBS). Cells were then permeabilized with 0.1% Triton X 100 in PBS for 1 h at room temperature, followed by blocking in 5% donkey serum in PBS tween 20 (0.1%) (PBST) for 1 h at room temperature. Then, the primary antibodies were added to each well in 0.2 mL volume and incubated at 4 °C overnight. The primary antibodies include rat anti-Ki67 (14-5698-82, 1:100 dilution, Thermofisher Scientific, Waltham, MA, USA) and mouse anti-COL1 (67288-1-Ig, 1:400 dilution, Proteintech, Rosemont, IL, USA). On the second day, the wells were washed with PBST 3 times for 5 min each. Then, goat-anti-rat-(IgG/IgM)-594 (Code: 112-585-068, Jackson ImmunoResearch Laboratories, West Grove, PA, USA) and donkey anti-mouse-IgG-488 (Code: 715-545-150, Jackson ImmunoResearch Laboratories) secondary antibodies at 1:200 dilution were added to each well and incubated at room temperature for 2 h. Wells were then washed 3 times, and nuclei were counterstained with DAPI for 30 min in PBST. Wells were finally washed with PBST 3 times for 5 min each and PBS 1 time for 5 min. For imaging, 1 mL PBS was added to each well. Fluorescent images were captured with Nikon ECLIPSE Ti microscope. COL1^+^ area percentage was quantified with NIS ELEMENT AR version software. Ki67^+^ cells were quantified with Image J (version 1.53K, Java 1.8.0_172), and Ki67^+^ cell percentage was calculated based on total DAPI^+^ nuclei.

#### 2.2.4. hBMP4 Enzyme-Linked Immunosorbent Assay (ELISA) of Cell Culture Supernatant

Supernatant collected at different time points from hBMMSCs treated with hBMP4 mRNA/LNP was centrifuged at 2000× *g* for 5 min prior to ELISA to remove particles. Human BMP4 protein secreted in the supernatant was measured with Quantikine™ ELISA Human BMP-4 Immunoassay (Cat#:DBP400, R&D system) following the manufacturer’s protocol. The A450 absorbance was measured with A540 correction with Tecan plate reader. The GraphPad Prism 10.2 software was used to make 4PL standard curve. The hBMP4 concentration was expressed as pg/mL.

### 2.3. Human BMP4 mRNA for Critical-Size Calvarial Defect Healing Using Fibrin Sealant as Scaffold

This experiment was approved by Institutional Animal Care and Use Committee (IACUC #3817) of Colorado State University. Briefly, 18-month-old C57BL6J mice were divided into three groups (*n* = 6–7, both males and females). Group 1: freezing medium (158 µL) + fibrinogen (30 µL) + thrombin (30 µL) scaffold; Group 2: 25 µg human BMP4 mRNA in 79 µL + 79 µL mRNA freezing medium + Fibrinogen (30 µL + thrombin (30 µL) combined and applied to the defect; Group 3: 50 µg mRNA in 158 µL + Fibrinogen (30 µL) + thrombin (30 µL). The creation of a critical-size 5 mm calvarial bone defect was performed as previously described [8]. Mouse skulls were shaved to remove fur. After sterilization with providine followed by 70% alcohol, an incision was made just off the midline of the scalp. The periosteum was removed, and a 5 mm critical-size defect was created with a 5 mm diameter trephine drill. Human BMP4 mRNA mixed with thrombin was then added to the defect using a 200 µL tip ring, and 30 µL of fibrin was immediately added. Hold the tip ring for 1–2 min to allow for fibrin to form a fibrin-sealant hydrogel in the defect area. The tip ring was then removed, and wounds were closed with sutures. Mice were recovered in a warm box and then transferred to their home cage.

#### 2.3.1. Live Micro-CT Scan to Monitor Bone Regeneration

Live micro-CT scanning was performed every two weeks as previously described [8] using viva-CT 80 (Scanco Medical LLC, Brüttisellen, Switzerland). The micro-CT scan parameters were 70 kVP, 110 µA and 30 µm voxel size. All micro-CT analyses were performed using the built-in micro-CT software V7.1–2. For the overview analysis of new bone formation in the defect area, we used the 400 × 200 dimension to define the defect area using Gauss Sigma = 0.8, Gauss support = 1 and threshold 163. New bone formation was quantified by manually contouring the new bone in each 2D image slice in combination with the morph function of the software to define new bone. New bone was mostly present at the edge of defect because of the presence of periosteum cells that hBMP4 mRNA/LNP can transfect and express BMP4. In 2D images, new bone and host bone can be clearly distinguished from one another, as new bone has irregular trabecular structure while host bone has dense bone microstructure. The new bone was defined using Gauss Sigma = 0.8, Gauss support = 1 and threshold 163.

#### 2.3.2. Bone Histology

Mice were sacrificed at 6 weeks after skull defect surgery following the last micro-CT scan. Skull tissue containing the defects was harvested, fixed in neutral buffered formalin (NBF) for 3 days, then decalcified with 10% EDTA plus 1% sodium hydroxide (PH = 7.0) for 4 weeks. The skull tissues were cut at the front ¼ of the defect and the middle of the defect for tissue processing and embedding. The tissues were placed in cassettes and processed using a Tissue Processor (KD-TS3D1, KEEDE) and paraffin-embedded in paraffin 9 (Fisher Scientific, Pittsburgh, PA, USA) using a tissue embedder (KD-BM-II, KEEDEE). Sections were then cut into 5 µm thickness using Leica Microtome (Histocore MULTICUT, Leica System, Wetzlar, Germany). Herovici’s staining was performed using a protocol as previously described [28,32] to differentiate collagen type 1 (COL1) and type 3 (COL3). Acid fuchsin (F8129-25G), picric acid (P6744-1GA), methyl blue (M5528-25g), acetic acid (Fisher Scientific) and ethanol (Fisher Scientific) were from Sigma-Millipore. H&E staining was performed using Extra-Strength Hematoxylin and Eosin Y (alcoholic) from ANATECH LTD following the manufacturer’s protocol. All slides were cover-slipped with Cytoseal mount medium (Fisher Scientific). Histological images were captured with Nikon CIS microscope using NIS BR software.

### 2.4. Human BMP4 mRNA/LNP for the Treatment of Natural Aged Mice Osteoarthritis

#### 2.4.1. Human BMP4 mRNA/LNP Intra-Articular Injection

This experiment was approved by IACUC of Colorado State University (Protocol#4839). For this experiment, 19-month-old C57BL6J mice including both males and females were divided into 3 groups: group 1 (CTL, *n*= 8), intra-articular injection of mRNA freezing medium in 15.8 µL; group 2 (*n* = 7), intra-articular injection of 2.5 µg lipid nanoparticle-encapsulated hBMP4 mRNA (total 15.8 µL including 7.9 µL mRNA freezing medium plus 7.9 µL original hBMP4 mRNA/LNP); group 3 (N = 8), intra-articular injection of 5 µg (15.8 µL) lipid nanoparticle-encapsulated hBMP4 mRNA. Intra-articular injections were performed in the left knee at baseline and repeated every three weeks for a total of 3 times. Mice were sacrificed 2 weeks after the last injection, and the knees were harvested and fixed in NBF.

#### 2.4.2. Pain Measurement

Mechanical sensitivity (pain threshold) was measured using a Von Frey device at baseline and at 4 and 8 weeks post-injection as previously described [33]. Briefly, mice were placed in the metal mesh box individually and acclimated for 30 min. Von Frey filaments were then applied to the plantar of the left paw. The filament was applied with enough force to elicit a withdrawal response. The stimulus intensity was recorded automatically by the device and documented immediately. This measurement was repeated 5 times for each mouse at 5 min intervals, and the average of the 5 measurements was used to represent the pain threshold.

#### 2.4.3. Micro-CT Scan and Analysis for Knee Joint

Mice were sacrificed at 8 weeks after IA injections of hBMP4 mRNA. The entire knee was scanned with Vivo-CT 80 using 70 kVp, 110 µA and 15 µm voxel size. The micro-CT was analyzed using built-in software version 6.6--5, and 3D images of the entire knee were reconstructed using Gauss Sigma = 0.8, Gauss support = 1, and threshold 163 to reveal heterotopic bone (HO) formation and general structural morphology of the knee as previously described [9].

#### 2.4.4. Histology

After the micro-CT scans, the tissues were decalcified with 10% EDTA plus 1% NaOH for 4 weeks, paraffin-embedded, and sectioned in 5 µm thickness as stated in Section 2.2.2. Alcian blue staining was performed using IHC world protocol as previously described [9] to detect acid mucin and hyaluronic acid. Alcian blue 8GX (Cat#: A5268-25g), nuclear fast red (cat#: N8002-5g), and aluminum sulphate (1003564536, Sigma-Aldrich, St. Louis, MO, USA) were purchased from Sigma. Acetic acid (Cat#: A38S-500) and ethanol (BP2828-4) were purchased from Fisher Scientific and freshly made before performing staining. Safranin O staining was performed using IHC world protocol with a slight modification to increase Safranin O step to 30 min to reveal the cartilage matrix glycosaminoglycans (GAGs). Safranin O (S2256-25g) and fast green FCF (F7252-5g) were purchased from Sigma. Weigert hematoxylin A (Cat#26102-1A) and B (Cat#:26102-1B) were purchased from Electronic Microscopy Science. Cartilage repair was evaluated using the Osteoarthritis Research Society International (OARSI) osteoarthritis histology score system [34] based on Safranin O and Alcian blue staining. Herovici’s and H&E staining were also performed as described previously [28] and as in Section 2.2.2.

#### 2.4.5. Immunohistochemistry

Immunohistochemistry (IHC) staining of type 2 collagen (COL2) and Sry-box transcription factor 9 (SOX9) was performed to reveal cartilage-specific matrix COL2 and SOX9. Briefly, paraffin sections were deparaffinized and rehydrated with deionized water. For COL2, slides were first subjected to antigen retrieval using 2% hyaluronidase in PBS for 30 min at room temperature (RT) as previously described [9]. Then, the slides were washed with PBS 3 times and blocked with a mouse-on-mouse (M.O.M) (BMK-2202, Vector Laboratories, Newark, CA, USA) blocking reagent in PBS for 1 h at room temperature. The slides were washed with PBS 2 times for 2 min and then incubated with M.O.M diluent for 5 min. Subsequently, the diluent solution was removed without washing, and 100 µL mouse anti-COL2 antibody (#7048, 1:500 dilution, Chondrex Inc., Woodinville, WA, USA) diluted in M.O.M diluent solution was applied and incubated at 4 °C overnight. For SOX9, no antigen retrieval was needed. After slides were deparaffinized and rehydrated with water, they were washed 3 times with PBS, then blocked with 5% donkey serum in PBS for 1 h at room temperature. Then, rabbit-anti-SOX9 antibody (PA5-81966, 1:500 dilution, Invitrogen/Thermofisher Scientific, Waltham, MA, USA) diluted with 5% donkey serum was applied to slides. On the second day, slides were washed with PBS 3 times for 5 min, and endogenous peroxidase was inactivated using 0.5% hydrogen peroxide in PBS for 10 min. Following another 3 washes with PBS, the slides were incubated with biotinylated horse anti-mouse secondary antibody (BA 2000, Vector Laboratories, Burlingame, CA, USA, 1:300 dilution) for COL2 and biotinylated horse anti-rabbit secondary antibody for SOX9 for 2 h at room temperature. After three washes, slides were further incubated with ABC reagent (PK 6100, VECTASTAIN^®^ Elite^®^ ABC-HRP Kit, Peroxidase (Standard), Vector Laboratories) for 2 h at room temperature. The slides were then washed 3 times with PBS, and diaminobenzidine (DAB) color reaction kit (SK-4100, Vector Laboratories) was used to visualize the COL2-positive cells and matrix or SOX9-positive cells. Hematoxylin (H-3404, Vector laboratories) counterstaining was performed after the DAB color reaction. Immunohistochemistry images were captured using a NIKON-CI microscope. Image J was used to quantify the SOX-9 positive cells in the entire cartilage and normalized to cells/200× field. The percentage of SOX9-positive cells in the entire cartilage was also calculated. Femoral condyle and tibial plateau cartilage were analyzed separately to be comparable.

#### 2.4.6. Serum Cartilage Damage Marker ELISA

Blood was collected at the time of sacrifice from the retro-orbital network and allowed to coagulate for 30 min. Serum was then isolated by centrifugation at 1500× *g* for 10 min and stored in a −80 °C freezer until analysis by ELISA. Hyaluronic acid (HA) levels were measured using a mouse hyaluronic acid (HA) ELISA Kit (Cat#:EK730393, AFG Scientific, Arlington Heights, IL, USA) following the manufacturer’s protocol. The standard curve was curve fit using a 4PL model, and serum concentration of HA was calculated from the 4PL curve using GraphPad Prism 10.2.

### 2.5. Statistical Analysis

All statistical analyses were performed using Graphpad Prism 10. Data was analyzed by ANOVA followed by Tukey’s multiple comparisons test, or Kruskal–Wallis non-parameter tests followed by Dunn’s multiple comparisons test if data was not normally distributed. The level of *p* < 0.05 was deemed statistically significant.

## 3. Results

### 3.1. hBMP4 mRNA In Vitro Transcription and Encapsulation Verification

**Figure 1:**
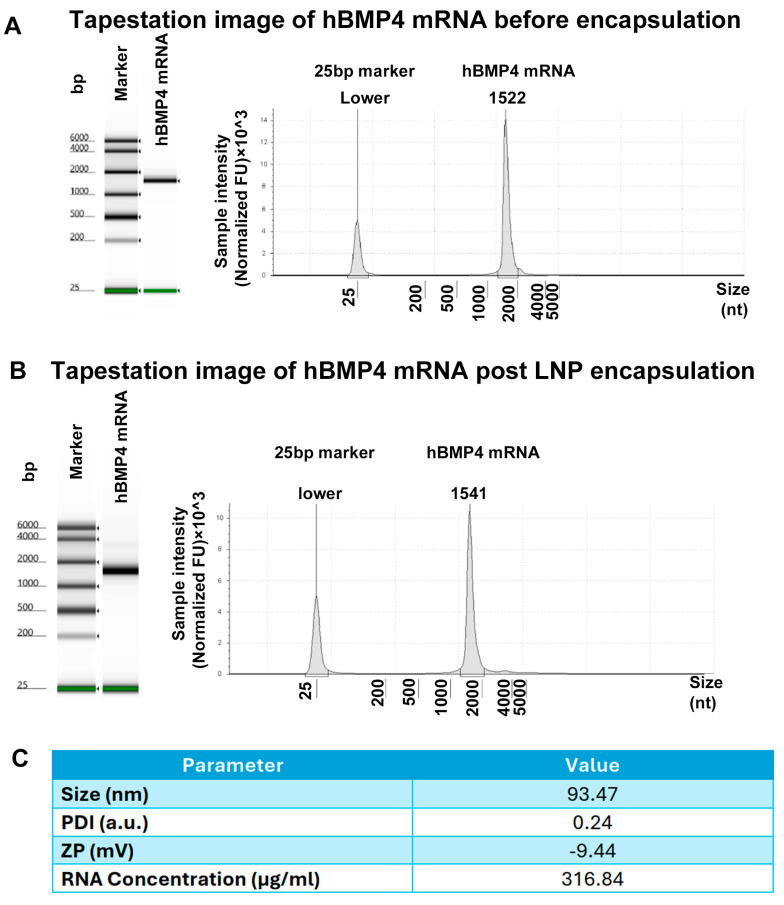
**hBMP4 mRNA in vitro transcription and encapsulation quality verification.** (**A**) hBMP4 mRNA band size measured by Tapestation. A 25 bp marker was added to samples to precisely determine the size of target mRNA, which can be viewed both on the gel and size graphs. (**B**) hBMP4 mRNA band size measured by Tapestation after LNP encapsulation. A 25 bp marker was added to samples to precisely determine the size of target mRNA which can be viewed both on the gel and size peak graphs. (**C**) hBMP4 mRNA/LNP particle size, PDI, zeta potential, and hBMP4 RNA concentration measurements performed with dynamic light scattering using Malvern Zetasizer.

A human BMP4 mRNA plasmid, purchased from Origene (Cat#:SC108985, NCBI Sequence ID: NM_110202, or NP_001193.1), was subcloned into a T7 promoter vector for in vitro transcription. In vitro transcription was performed by RNAcore from Houston Methodist Academic Institute. To generate chemically modified hBMP4 mRNA, in vitro transcription incorporated N1-methyl pseudo-urindine triphosphate (N1-M-pUTP) with CleanCap. The full size of transcribed hBMP4 mRNA is 1603 bp. After purification of hBMP4 mRNA, the quality was verified using Tapestation (Figure 1A). The hBMP4 mRNA was subsequently encapsulated with SM-102 lipid nanoparticles. The final hBMP4 mRNA/LNP quality met the quality requirement for particle size, polydispersity index (PDI), and zeta potential (ZP), with a final concentration of 316 µg/mL after encapsulation (Figure 1B,C).

### 3.2. hMSCs Transfected with hBMP4 mRNA/LNP Secreted hBMP4 Protein and Underwent Enhanced Osteogenic Differentiation

**Figure 2:**
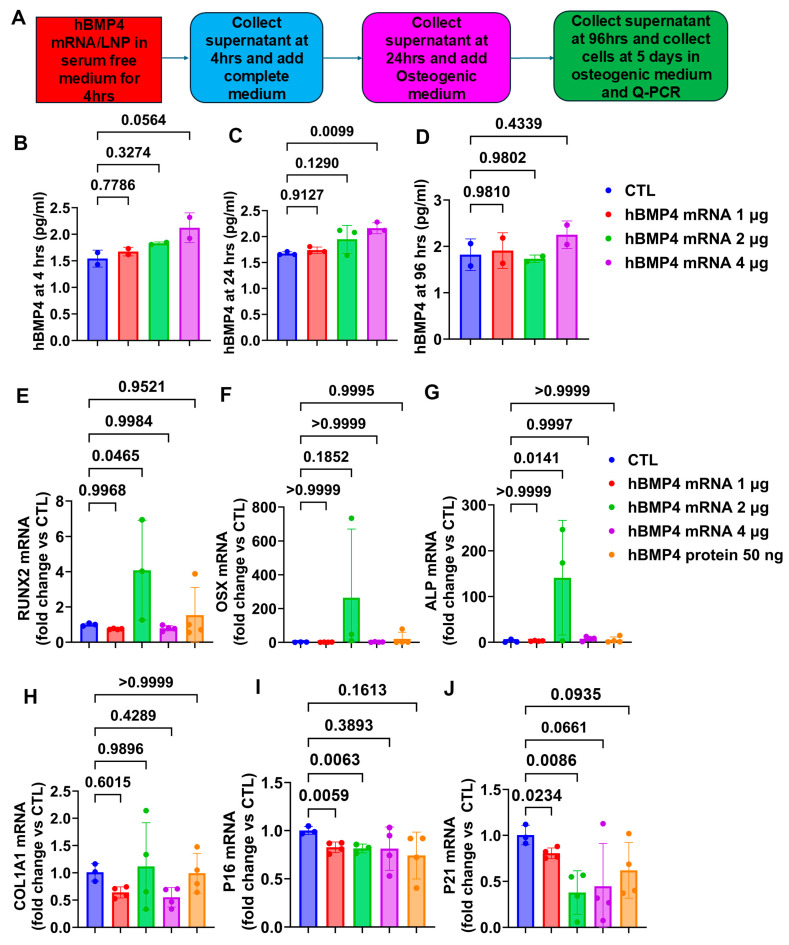
hBMMSCs transfected with hBMP4 mRNA/LNP secreted hBMP4 protein and enhanced osteogenic differentiation. (**A**) Schematic of experiment design. (**B**–**D**) hBMP4 protein ELISA at different time points. BMP4 secretion dose-dependently increased in the supernatant at 4 h, reaching peak expression 24 h, with the 4 µg group being significantly increased compared to CTL. Expression declined at 96 h after transfection. (**E**–**H**) Q-PCR analysis of osteogenic genes’ mRNA after 5 days of osteogenic differentiation. (**I**,**J**) Q-PCR analysis of senescence/cell cycle genes P16 and P21. Exact *p* values are indicated between group bars.

To test if hBMP4 mRNA/LNP are functional in vitro, hMSCs were transfected with HBMP4 mRNA/LNP for 4 h at different doses. A slight increase was observed in hBMP4 protein level at 4 h post-transfection (Figure 2B), with the 4 µg group having a nearly statistically significant increase (*p* = 0.0564). By 24 h after changing the medium, BMP4 secretion increased in a dose-dependent manner, reaching statistical significance at the 4 µg dose. By 96 h post-transfection, BMP4 secretion in the 4 µg treatment group was still higher than in the CTL group, but not reached statistically significance. As a positive control for the ELISA, the hBMP4 protein group showed the highest A450, which exceeded the reading limit of the plate reader (Figure 2C,D). These results indicate that hBMP4 mRNA/LNP mediated hBMP4 protein expression in the cells. Furthermore, Q-PCR analysis of osteogenic gene expression after 5 days revealed a significant increase in RUNX2 (Figure 2E) and ALP expression (Figure 2G) in the 2 µg group (4-fold and 140-fold increase, respectively), while OSX also showed a 263-fold increase (Figure 2F) in this group despite not reaching statistical significance due to high variation. COL1A1 was highly expressed, as demonstrated by lower CT values, but there was no significant increase compared to the CTL group. Surprisingly, hBMP4 protein at 50 ng/mL also did not significantly increase osteogenic gene expression (Figure 2H). Furthermore, when we examined P16 and P21 expression, we found that P16 mRNA expression was significantly decreased at the 1 µg and 2 µg doses and showed a decreasing trend in the 4 µg dose and hBMP4 protein groups (Figure 2I). Furthermore, we found that P21 mRNA expression was also significantly downregulated in the 1 µg and 2 µg dose, and a decreasing trend was observed in the 4 µg and hBMP4 protein groups (Figure 2J).

**Figure 3:**
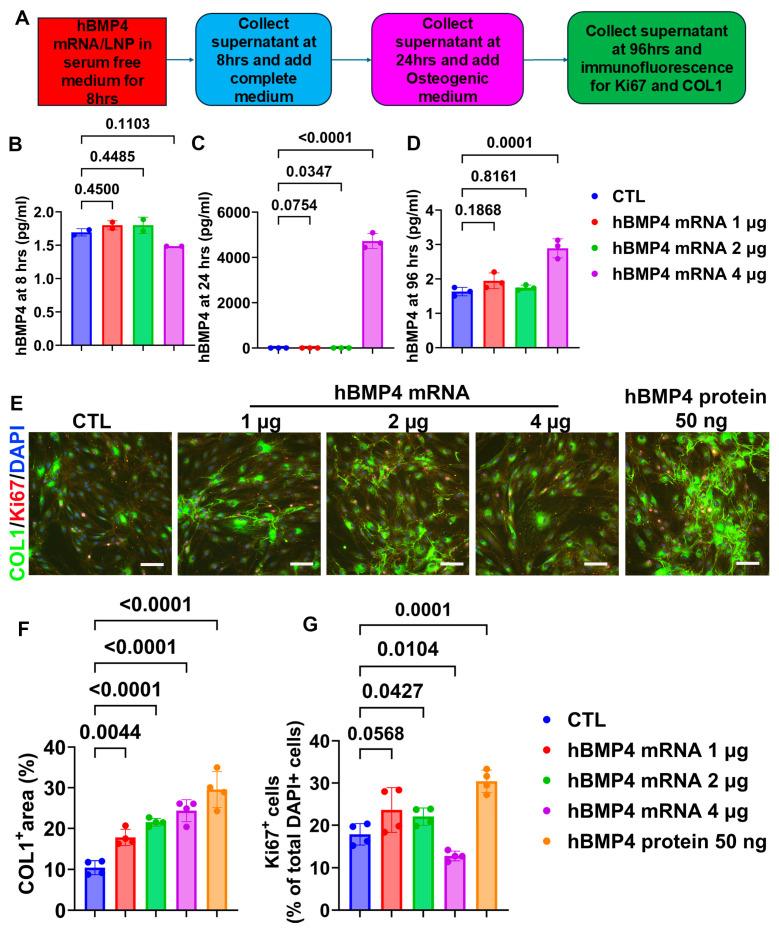
Human BMP4 secretion and immunofluorescence staining of Ki67 and COL1 after 8 h of transfection and 5 days of osteogenic differentiation. (**A**) Schematic of the experiment design. (**B**–**D**) hBMP4 secretion level. (**E**) Immunofluorescence staining representative images of Ki67 (red) and COL1 (green). Scale bars = 100 μm. (**F**) Quantification of COL1^+^ area percentage. (**G**) Quantification of Ki67^+^ cell percentage. Exact *p* values are indicated between group bars.

To test if increasing the transfection time would increase hBMP4 mRNA expression, we transfected the hBMMSCs for 8 h at the same doses (Figure 3A). There was no obvious cell death after the 8 h transfection period, as observed by microscopy immediately after transduction and during the cell recovery and osteogenic differentiation process. hBMP4 ELISA results demonstrated no significant increase in BMP4 secretion at any dose 8 h after transfection (Figure 3B). However, at 24 h post-transfection, BMP4 secretion showed an increasing trend in the 1 µg dose (*p* = 0.0754) (Figure 3C), while there was a significant increase in BMP4 secretion in the 2 µg dose (*p* = 0.0347) (Figure 3C). Strikingly, BMP4 secretion was dramatically increased (4724 pg/mL vs. CTL 1.69 pg/mL) (Figure 3C). At 96 h post-transfection, BMP4 secretion remained significantly higher than in the CTL group (Figure 3D). These results indicate that increasing the transfection time could increase target hBMP4 mRNA transfection and subsequently increase protein expression and secretion. Furthermore, we performed immunofluorescence staining for osteogenic genes COL1 and cell proliferation for Ki67. We found that hBMP4 mRNA dose-dependently increased COL1^+^ area percentage, with all doses being significantly higher than in the CTL group despite the intensity in the 4 µg group being relatively low (Figure 3E,F). These results indicate that hBMP4 mRNA/LNP is functional, but they are all lower than those for the group with hBMP4 protein at 50 ng/mL. We also quantified Ki67 to detect cell proliferation and found that hBMP4 mRNA at 1 and 2 μg increased Ki67^+^ cell percentage, with 2 μg being significantly increased relative to the CTL group. However, in the 4 μg group, Ki67^+^ cell percentage significantly decreased, which might indicate the dose is too high. Positive-control hBMP4 protein also significantly increased Ki67^+^ cell percentage and was higher than any other hBM4 mRNA group.

### 3.3. HBMP4 mRNA Promoted Limited Bone Regeneration in a Critical-Size Calvarial Bone Defect in Aged Mice

**Figure 4:**
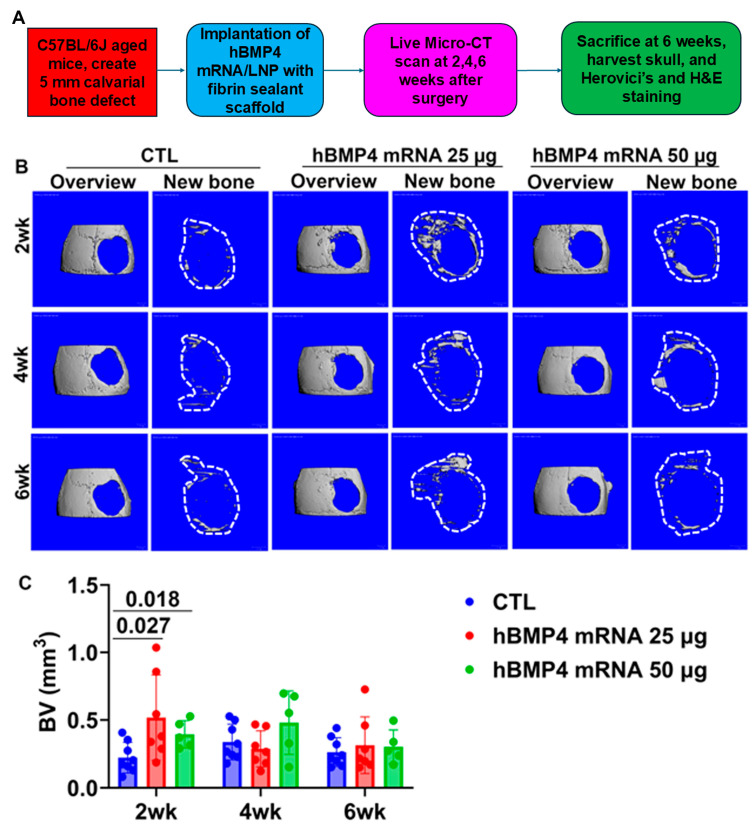
3D micro-CT live scan images to monitor bone defect healing after implantation of hBMP4 mRNA. (**A**) Schematic experiment design and timeline. (**B**) 3D micro-CT overview of the entire defect and segmentalized new bone formation at 2, 4, and 6 weeks in different groups: Minimal new bone formation was detected at the edge of the critical-size calvarial bone defect in the control group. Relatively higher amount of new bone formation in the 25 µg and 50 µg hBMP4 mRNA groups was observed at all time points. However, complete defect healing was not observed in any group by 6 weeks. New bones are circled in dashed white lines, segmented from the skull host bone. (**C**) Quantification of new bone formation in the defect area: new bone volumes are significantly higher in the 25 µg and 50 µg groups at 2 weeks after application of hBMP4 mRNA, but no significant differences were found at 4 and 6 weeks after application. Exact *p* values are indicated between group bars.

Following application of hBMP4 mNRA/LNP using a fibrin sealant scaffold immediately after creation of a critical-size calvarial bone defect, we monitored the bone formation using live micro-CT scans at 2, 4 and 6 weeks post-surgery. A 3D micro-CT overview of the entire defect and segmentalized new bone formation demonstrated minimal new bone formation at the edge of the critical-size calvarial bone defect in the control group. A relatively higher amount of new bone formation was observed in the 25 µg and 50 µg hBMP4 mRNA groups at all time points. However, complete defect healing was not observed in any group by 6 weeks (Figure 4A). Quantification of the new bone formation in the defect area revealed significantly higher new bone volumes in the 25 µg and 50 µg groups at 2 weeks after the application of hBMP4 mRNA, but no significant differences were found at 4 and 6 weeks after application compared to the control group (Figure 4B).

### 3.4. Histology Revealed Minimal New Bone Formation Mediated by hBMP4 mRNA/LNP in Critical-Size Calvarial Bone Defect When Delivered with Fibrin Sealant

**Figure 5:**
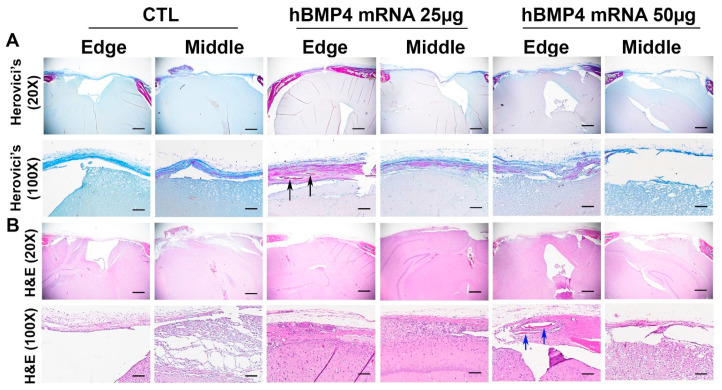
Histology of the skull defect after hBMP4 mRNA application. (**A**) Herovici’s staining to reveal new bone formation: COL1 stained in pink-red, and COL3 stained in dark blue. No new bone formation was revealed in the defect area in the CTL group at either the edge or in the middle of the defect. Only blue-stained COL3^+^ scar tissue was observed. In the 25 µg group, only island bone was detected at the edge of the defect (black arrows). No new bone was detected in the middle of the defect. No new bone formation in the defect area was found at this section level for the 50 µg hBMP4 mRNA group. (**B**) H&E staining: No new pink-stained new bone was detected at the edge or in the middle of the defect area for the CTL and 25 µg hBMP4 RNA groups. Only island bone was detected at the edge of the defect of the 50 µg hBMP4 mRNA group, as indicated by blue arrows. Scale bars = 500 µm for 20× and 100 µm for 100×.

We performed Herovici’s staining to detect COL1 (a major bone matrix) and COL3, a fibrotic collagen. At 20×, the entire defect view was revealed at ¼ of the defect (edge) or the middle of the defect. The residual host bone is evident on both edges of the defect for all groups, with intense pink-red staining. The CTL group showed no bone formation either at the edge or in the middle of the defect. In the 25 µg group, some new, island-like bone was detected at the edge of the defect. No pink-stained COL1 was detected in the middle of the defect. In the HBMP4 50 µg group, no pink-red COL1 was detected either at the edge or in the middle of the defect. At 100×, in the CTL group, only blue-stained COL3 was detected either at the edge or in the middle of the defect. Pink-stained COL1 was detected at the edges of the defect of the HBMP4 mRNA 25 µg group (black arrows) but not in the middle of the defect. No pink-stained COL1 was detected at the edge or in the middle of the defect in the hBMP4 mRNA 50 µg group (Figure 5A). H&E staining revealed dense trabecular residual host bone. The defect was filled with fibrotic tissue in the CTL group. There was also no new bone formation in the hBMP4 25 µg group at the edges or in the middle of the defect. At the edge region, a small bone island of dense tissue was detected (blue arrows), and only fibrotic tissue in the middle of the defect was found in the 50 µg hBMP4 mRNA/LNP group. The new bone did not have a trabecular bone structure (Figure 4B).

### 3.5. Micro-CT Results Revealed No Increase in Heterotopic Bone Formation in the Knee Joint at 8 Weeks After Intra-Articular Injection of BMP4 mRNA

**Figure 6:**
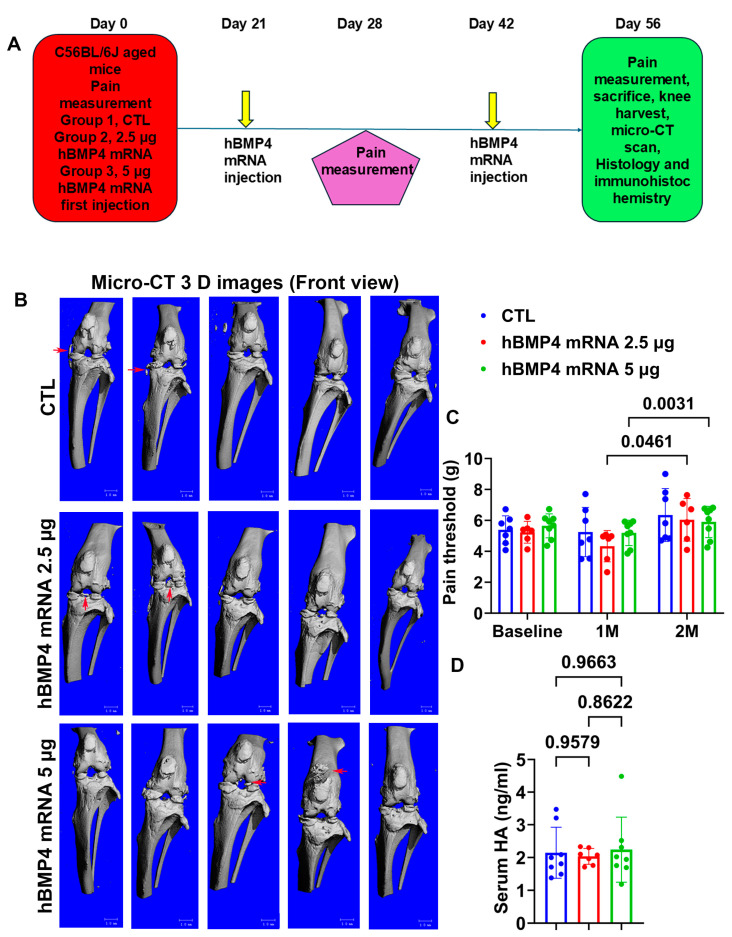
Micro-CT results of knee joint at 8 weeks after BMP4 mRNA injection. (**A**) Schematic experiment design and timeline. (**B**) 3D micro-CT images of the knee. No increase in heterotopic bone formation was observed in BMP4 mRNA at 2.5 µg and 5 µg doses compared to CTL group. Red arrows point to HO formation. The slight differences in image sizes are due to differences in total slice numbers as well as knee angle during micro-CT scanning. Scale bars = 1 mm. (**C**) Von Frey pain threshold. There were no statistical differences at baseline and 1 M after mRNA injection. At 2 M, both BMP4 mRNA 2.5 and 5 µg had increased pain thresholds compared to 1 M after injection, which indicated reduced pain. Exact *p* values are indicated between group bars. (**D**) ELISA results of serum HA. Exact *p* values are indicated between group bars.

The experiment design is as shown in Figure 6A. 3D micro-CT images revealed a mild heterotopic ossification (HO) formation (red arrows) in each group, which was likely caused by mild injury due to injection or age-related osteophyte formation. HO or osteophyte formation is one of the common pathological changes associated with age-related OA or traumatic arthritis [35]. HBMP4 mRNA at 2.5 and 5 µg doses did not increase the rate of HO formation. The large HO formation in one sample from the hBMP4 mRNA 5 µg group was likely due to leakage of the injected hBMP4 mRNA into the muscle (Figure 6B).

### 3.6. hBMP4 mRNA Intra-Articular Injection Appeared to Alleviate Pain Threshold Measured by Von Frey Device

We also measured pain using a Von Frey device before injection and again at 1 and 2 months after injection. The pain threshold was assessed using a Von Frey device, which measures the paw withdrawal response threshold to applied force using sharp filaments. A higher threshold of applied force before paw withdrawal means less sensitivity to pain. Our results revealed no significant difference between groups at baseline, but the pain threshold increased at 1 month and 2 months after injection in the 2.5 and 5 µg hBMP4 mRNA groups after IA injection (Figure 6C). These results indicate that IA injection of hBMP4 mRNA/LNP may alleviate the pain of age-related natural osteoarthritis.

### 3.7. Intra-Articular Injection of hBMP4 mRNA Did Not Significantly Change Serum Cartilage Damage Marker Hyaluronic Acid (HA)

We further performed an ELISA analysis of serum hyaluronic acid (HA). We found no significant differences among different groups (Figure 6D).

### 3.8. hBMP4 mRNA/LNP Intra-Articular Injection Prevented Age-Related Cartilage Loss and Improved Histology Score

**Figure 7:**
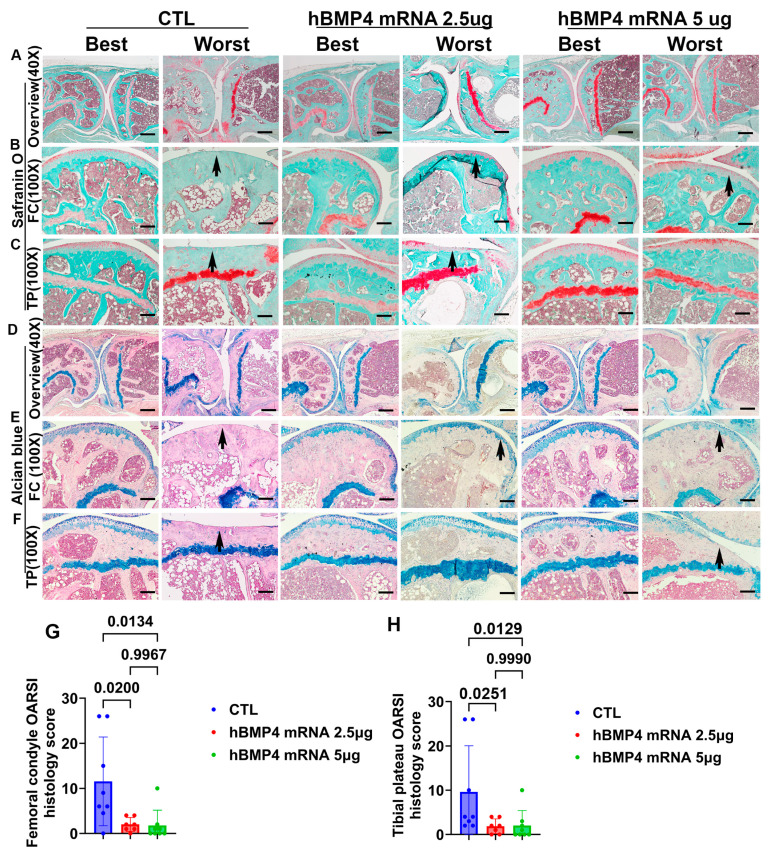
Aged cartilage histology after IA injection of hBMP4 mRNA/LNP. (**A**–**C**) Safranin O staining of entire knee joints (40×), femoral condyle and tibial plateau cartilage. Best and worst knee repairs are shown for all groups. Cartilage or chondrocyte GAGs stained orange-red, nuclei stained black and cytoplasm stained green. (**D**–**F**) Alcian blue staining of entire knee, femoral condyle and tibial plateau. Acid mucin and HA stained blue. Nuclei are stained red and cytoplasm is stained pale pink. (**G**) Femoral condyle OARSI histology score. (**H**) Tibial plateau histology score. Black arrows indicate different extents of cartilage damage from complete loss of cartilage (denuding) to mild fibrillation. Scale bars = 250 µm for 40× and 100 µm for 100×. Exact *p* values are indicated between group bars.

To evaluate whether hBMP4 mRNA/LNP can improve age-related osteoarthritis, Safranin O staining was performed. The best and worst cartilage OA repair is shown for all groups. The entire medial side of the knee joint structure is displayed, with the femur on the left side and the tibia on the right side (Figure 7A). The CTL group had three mice with severe cartilage damage, and the mouse with the worst repair had no orange-red-stained cartilage in the articular cartilage surface (denuding), as demonstrated in both the femoral condyle and tibial plateau cartilage (Figure 7B,C, black arrows). Both the 2.5 and 5 µg hBMP4 mRNA groups had only one mouse with severe cartilage damage, with partial cartilage loss and complete loss of the orange-red matrix. Other mice from each group demonstrated relatively mild or even normal cartilage morphology (Figure 7A–C). No heterotopic bone or osteophyte formation was identified on the articular surface. Alcian blue staining labeled cartilage matrix acid mucins and hyaluronic acid in blue, revealing a complete loss of blue-stained matrix in the worst-repaired joint of the CTL group. By contrast, hBMP4 mRNA/LNP-injected mice had only partial cartilage loss in the worst case. No abnormal bone structure was found on the articular surface (Figure 7D–F). Furthermore, the OARSI histology score of the medial femoral condyle was significantly lower (better repair) in the two hBMP4 mRNA groups when compared to the CTL group, but there were no differences between the two hBMP4 mRNA groups (Figure 7G). The OARSI histology score of the medial tibial plateau cartilage was also significantly lower in the two hBMP4 mRNA dosage groups compared to the CTL group, with no difference observed between the two hBMP4 mRNA groups (Figure 7H).

### 3.9. hBMP4 mRNA/LNP Intra-Articular Injection Maintained Cartilage Matrix During Aging

**Figure 8:**
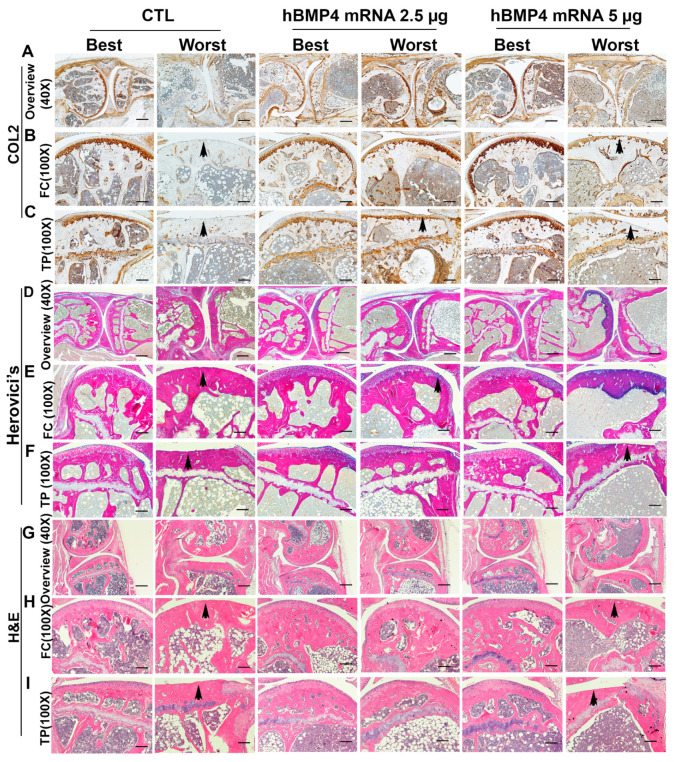
**COL2 IHC, Herovici’s staining, and H&E staining at 8 weeks after IA hBMP4 mRNA injection.** (**A**–**C**) Immunohistochemistry of COL2 for entire knee, femoral condyle and tibial plateau. COL2 is stained brown in chondrocytes and cartilage matrix as well as residual expression in subchondral bone. (**D**–**F**) Herovici’s staining for entire knee, femoral condyle and tibial plateau. COL1 is stained in pink-red, and COL3 stained in dark blue. (**G**–**I**) H&E staining to reveal general morphology of articular cartilage. Nuclei are stained blue and cytoplasm is stained red. Black arrows indicate different extents of cartilage damage, from complete loss of cartilage (denuding) to mild fibrillation. Scale bars = 250 µm for 40× and 100 µm for 100×.

We performed immunohistochemistry for cartilage-specific matrix COL2. COL2 was stained brown both inside chondrocytes and in the extracellular matrix of the cartilage. COL2 was completely lost in the CTL group’s worst repair, while there was only partial loss in the worst repair of the two hBMP4 mRNA dosage groups. Focal COL2-positive cells were detected in the proliferation zone of the two hBMP4 mRNA groups (Figure 8A–C). Herovici’s staining revealed no new pink-stained bone structure in the joint at lower magnification. Mixed blue and red staining was found in the best-cartilage-repair animal of each group, while only pink COL1 was present in the worst repair of the CTL group, and the two hBMP4 mRNA groups showed partial cartilage loss (Figure 8D,E). H&E staining revealed a typical morphology in the animal with the best cartilage repair and only subchondral bone in the worst repair of the CTL group. By contrast, there was only a loss of partial cartilage cells in the worst repair of the two hBMP4 mRNA groups (Figure 8G,H).

### 3.10. hBMP4 mRNA/LNP IA Injection Enhanced SOX9 Expression in the Knee Joint Cartilage

**Figure 9:**
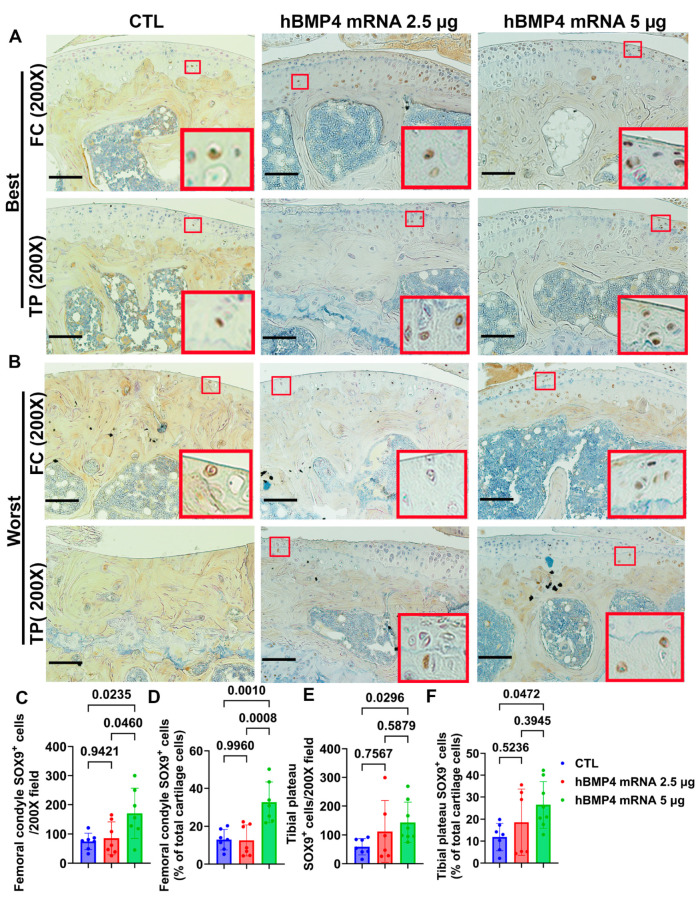
**Immunohistochemistry of SOX9 and quantification.** (**A**) Representative SOX9 immunohistochemistry images of best cartilage repair of femoral condyle and tibia plateau in each group. Nuclei of SOX9-positive cells are stained brown. Insets highlight positive cells. (**B**) Representative SOX9 immunohistochemistry images of the worst cartilage repair in femoral condyle and tibial plateau in each group. Insets highlight positive cells. Scale bars = 100 μm. (**C**,**D**) Quantification of SOX9^+^ cells/200× field and SOX9^+^ cell percentage of the entire cartilage of femoral condyle. (**E**,**F**) Quantification of SOX9^+^ cells/200× field and SOX9^+^ cell percentage of the entire cartilage of tibial plateau. Exact *p* values are indicated between group bars.

We further performed immunohistochemistry for SOX9, a master transcription factor of cartilage. SOX9-positive cells were detected throughout the entire cartilage. There were fewer SOX9-positive cells detected in the CTL group in the best cartilage repair in the femoral condyle or tibial plateau. The nuclei of SOX-9 positive cells were stained brown (highlighted in insets) and mainly located within the hypertrophic cartilage layer, whereas positive staining in the hBMP4 mRNA groups was also detected in the superficial and proliferative zones (Figure 9A). In the worst-repaired cartilage, nearly no cartilage and no positive cells were observed in the CTL group. Few positive cells were detected in the hBMP4 mRNA 2.5 μg group. More positive cells (highlighted in insets) were found in both the cartilage and hypertrophic cartilage layers in the hBMP4 mRNA 5 μg group (Figure 9B). Further quantification demonstrated that the SOX9^+^ cells/200X field and SOX9^+^ cell percentage in the femoral condyle increased significantly in the hBMP4 mRNA 5.0 μg group compared to the CTL and hBMP4 mRNA 2.5 μg groups (Figure 9C,D). Furthermore, SOX9^+^ cells/200X field and SOX9^+^ cell percentage in the tibial plateau were also significantly higher in the hBMP4 mRNA 5 μg group compared to the CTL group (Figure 9E,F). These results indicate that hBMP4 mRNA likely promotes cartilage repair in aged mice via increasing SOX9 expression.

## 4. Discussion

In this study, we successfully manufactured hBMP4 mRNA and encapsulated it in LNPs. hBMP4 mRNA/LNP-transfected hBMMSCs secreted hBMP4 protein transiently and promoted osteogenic differentiation in the transfected cells. However, in vivo, we found that hBMP4 mRNA/LNP at 25 µg and 50 µg dosages promoted a relatively higher amount of bone formation than the control only at an earlier time point after the creation of a bone defect when delivered using fibrin sealant hydrogel. The new bone formation did not increase further compared to the control at later time points, as revealed by micro-CT and histology. However, IA injection of hBMP4 mRNA/LNP at 2.5 µg and 5 µg doses into the knees of aged mice improved cartilage repair by decreasing the OARSI histology score of the articular cartilage of both the femoral condyle and tibial plateau, as demonstrated by Safranin O and Alcian blue staining as well as by immunohistochemistry staining of collagen type 2 (COL2). hBMP4 mRNA/LNP also increased the number and percentage of SOX9^+^ cells in both the femoral condyle and tibial plateau. The hBMP4 mRNA injection did not increase HO formation in the knee compared to the CTL injection group.

BMPs are a group of the most potent bone growth factors for bone tissue engineering [8] and have been approved by the FDA for several clinical applications [36]. Following the successful use of the COVID-19 mRNA vaccine to control the COVID-19 pandemic, several studies have reported the use of mRNA/LNP as a delivery approach to generate therapeutic proteins in the host cells to promote bone regeneration. Elangovan S et al. first demonstrated that chemically modified ribonucleic acid (cmRNA) encoding BMP-2 delivered using polyethylenimine (PEI) increased BMP-2 protein expression in transfected human bone marrow mesenchymal stem cells (hBMMSCs) compared to PEI-BMP2 pDNA-transfected hBMMSCs at the same dose. When implanted into rat calvarial bone defect, the PEI-cmRNA encoding BMP-2 (25 µg BMP-2 cmRNA) polyplexes regenerated more bone in the defect, with higher bone volume (BV/TV) compared to 25 µg BMP-2 pDNA polyplexes and significantly higher BV/TV than the empty defect group. Histological results showed more mature mineralized bone tissue in the BMP-2 PEI-cmRNA group, compared to minimal new bone formation at the edge of the defect in the pDNA group [37]. Surisaeng T et al. showed that BMP-2 cmRNA/LNP loaded on either silk fibroin (SF) or gelatin silk fibroin (G) scaffolds increased both the amount of mineralized bone matrix in the defect and the percentages of calvarial defect closure, as well as BV/TV, compared to empty defects. When using the G scaffold, all BMP-2 mRNA doses (1.5, 5, and 15 µg) significantly increased BV/TV, while when using the SF scaffold, only the 15 µg dose of BMP-2 mRNA showed a statistically significant increase in BV/TV, which indicates that the G scaffold is more suitable for the delivery of BMP2 cmRNA. However, only less than 60% defect closure was observed in both scaffold groups [38]. Furthermore, Khorsand B et al. also compared the bone regeneration potential of nanoplexes of polyethylenimine (PEI)-delivered BMP-9 cmRNA (10 µg) and BMP-9 pDNA (10 µg) loaded into perforated collagen membranes (PCMs) using a critical-size calvarial defect model in rats. It was found that PEI-delivered cmRNA encoding BMP-9 loaded on PCMs significantly enhanced new bone formation and BV/TV compared to the scaffold-only group, and a non-significant increase in BV/TV was observed compared to the PEI-pDNA-PCM group [25]. The same group of authors also compared cmRNA encoding BMP-2 and BMP-9 complexed with PEI at a 50 µg dose. When loaded on collagen scaffolds, freeze-dried, and implanted into the 5 mm calvarial bone defect in rats, both BMP-2 and BMP-9 cmRNAs regenerated significantly more bone compared to the empty defect (control) group, with complete defect closure, while the control group largely had no bone formation [39].

BMP2 mRNA has also been investigated for long bone defect healing. Balamayor E et al. reported that BMP-2 cmRNA-transfected stem cells demonstrated enhanced osteogenic differentiation in vitro. When BMP-2 cmRNA (2.5 µg) loaded on Fibrin + C12-EPE/hBMP-2 cmRNA lipoids was subsequently implanted into a 3 mm non-critical-size rat femoral defect model, more mineralized callus bone was formed at 2 weeks compared to the fibrin-only control defect. At later time points, more mature bone was observed in the BMP-2 cmRNA group, as revealed by histology [40]. Furthermore, Badieyan ZS et al. developed vacuum-dried, cmRNA-loaded collagen sponges, termed transcript-activated matrices (TAMs). BMP-2 cmRNA at 2.5 µg loaded on TAMs applied to a 2 mm non-critical drill-hole rat femur defect significantly increased bone callus volume, bone area, and osteoid volume, as confirmed by μCT and histomorphometric analysis [41]. De La Vega RE et al. compared the bone regeneration of BMP-2 cmRNA with recombinant BMP-2 protein (rhBMP-2) in a critical-size rat femoral defect model. Different doses (12.5 μg to 50 μg) of BMP-2 cmRNA or rhBMP-2 protein were loaded on a collagen sponge and implanted into a 5 mm femur defect covered with a deep muscle pouch. It was found that BMP-2 cmRNA promoted bone repair in a dose-dependent manner, with complete defect bridging found at 50 μg but not at lower doses. The 50 μg cmRNA group also showed greater BV/TV and Tb.N at 8 weeks after surgery, which indicates faster mechanical recovery and bone remodeling than the control group, and produced superior bone quality when compared with all other groups. Similar results were observed with the application of 11 µg rhBMP-2 [24].

Previously, we have demonstrated that retro-BMP4GFP-transduced murine MDSCs efficiently regenerated bone in calvarial bone defects and completely healed bone defects in 4 weeks when MDSCs secreted about 19 ng/million cells/24 h [42]. We also demonstrated that BMP4 can improve the osteogenic potential of aged MDSCs and promote bone formation in aged MDSCs via the regulation of cell cycle inhibitors such as P16 [22], but no study has been conducted using human BMP4 mRNA for calvarial bone defect repair. Therefore, we chose chemically modified BMP4 mRNA for this study. However, even high doses of hBMP4 mRNA (50 µg/5 mm defect) did not significantly promote bone defect healing in aged mice in the current study. This contrasts with what we have accomplished using coacervate sustained-release biomaterials with rhBMP4 protein. Only 2 µg of rhBMP4 protein can generate more bone than 50 µg of mRNA using the same fibrin sealant scaffold [8], and it is negligible compared to BMP4-transduced MDSCs, which generated more than 100 mm^3^ of new bone using a fibrin sealant scaffold, with complete healing in 4 weeks [42]. The reason for the inefficiency of hBMP4 mRNA/LNP might be that we used fibrin sealant hydrogel as scaffold, which is hydrophilic, while the LNP is hydrophobic. Other delivery vehicles or scaffolds such as collagen sponge may be more compatible with hBMP4 mRNA/LNP [24,43]. The other reason might be that we applied the BMP4 mRNA immediately after the defect creation, meaning there were very few cells present for BMP4 mRNA to transfect to express the target proteins. The third reason might be that most reported chemically modified mRNA can only express target proteins within 1 week of administration. Therefore, it might be more effective when applied at 1 week after injury, when host repairing cells such as MSCs have already migrated to the defect area. Previous studies using cmBMP2 mRNA also resulted in only partial defect closure in a calvarial bone defect model when using doses of 25 µg or less [25,37,38]. Only 50 µg cmBMP2 or BMP9 mRNA/PEI loaded on collagen scaffolds results in complete defect closure [39]. Therefore, a scaffold that maintains BMP cmRNA in the defect until cells have migrated into the defect is also important. Overall, cmRNA required a much higher dose to achieve similar regenerative effects compared to proteins, as demonstrated by other researchers and by us.

Osteoarthritis is a chronic disease that involves many factors, including inflammation, loss of chondrocytes and cartilage matrix, activation of cartilage-degrading enzymes, and osteophyte formation [44]. Current tissue engineering approaches for cartilage repair include targeting inflammation [45], the use of stem cells [46], and cartilage growth factors [47,48,49,50], with the goal of regenerating cartilage, restoring function, and relieving pain. Currently, there is no disease-modifying therapy available. Therefore, it is critical to develop novel therapeutics for age-related osteoarthritis.

Several studies have investigated cmRNA for cartilage repair. In 2016, Aini L et al. developed two polyethylene glycol (PEG)-polyamino acid block copolymer-based polyplex nanomicelles called PEG-PAsp(DET) and PEG-PAsp(TET) to deliver runt-related transcription factor 1 (Runx1) cmRNA. Intra-articular injection of Runx1 mRNA (1 µg in 20 µL volume every 3 days for one month) via PAsp(DET) nanomicelles non-significantly improved medial collateral ligament (MCL)- and medial meniscus (MM)-transection-induced OA compared to GFP mRNA. By contrast, delivery of the same dose of Runx1 mRNA using PEG-PAsp(TET) nanomicelles in the same OA model induced Runx1 expression in both the superficial and middle zones of the articular cartilage, significantly decreased OARSI histology score and inhibited osteophyte formation, and upregulated SOX9 and COL2 expression and cell proliferation in the cartilage [51]. Furthermore, Pezzotti G et al. used Raman spectroscopic analysis and demonstrated that the mechanism of cartilage restoration was attributed to the activation of the remaining chondrocytes by Runx1 cmRNA, which resulted in increased hyaluronic acid synthesis and the restoration of organized collagen secondary structures [52]. An additional study demonstrated that intra-articular injection of IGF-1 mRNA-transfected adipose-derived stem cells (ADSCs) (2 × 10^5^) at 1 and 2 weeks post-DMM surgery ameliorated articular cartilage degeneration, as revealed by decreased OARSI histology score and increased COL2 and ACAN expression [53].

Several studies also used fibroblast growth factor 18 (FGF18) mRNA for cartilage repair. Huang K et al. developed a new lipid nanoparticle TG6A with branched tails and five ester bonds, which demonstrated a higher efficiency of delivery compared to commercialized DLin-MC3-DMA and ALC-0315 lipid nanoparticles. MSCs transfected with TG6A LNP-encapsulated circular FGF18 mRNA enhanced chondrogenic differentiation in an in vitro 3D pellet culture model. Circular FGF18 cmRNA-engineered MSCs improved cartilage repair in a rat OA model, as revealed by thicker cartilage layers, reduced histopathological scores, the maintenance of zone structure, and higher type II collagen and extracellular matrix (ECM) deposition compared to an untreated control [54]. Sun M et al. designed a novel articular cavity-localized lipid nanoparticle (LNP) named WG-PL14 that could increase mRNA expression approximately 30-fold and was enriched in the articular cavity compared with commercial MC3 lipids after intra-articular injection. Intra-articular injection of 2 µg (in 20 µL/knee, once a week for 3 weeks) cmRNA encoding FGF-18 complexed with WG-PL14 LNPs delayed OA progression in a ACLT-induced OA model, as demonstrated by increased cartilage extracellular matrix (ECM)-related genes such as COL2 and aggrecan (ACAN) and decreased COL1, MMP13 and IL1β. FGF18 cmRNA also decreased osteophyte formation and increased tibial subchondral bone BV/TV, Tb.Th bone mineral density and improved pain response [55]. Kong K et al. also showed that LNP/FGF18 mRNA can penetrate deeper into cartilage layers than proteins. Intra-articular injection of FGF18 mRNA/LNP (6 µg weekly in 20 µL) or 500 ng FGF18 protein (in 20 µL) for 8 weeks in the joint of DMM-induced and age-related OA models improved OA phenotype through the activation of the FOX3A-autophage pathway, decreasing degeneration and senescence [56]. cmRNA has also been used for osteochondral defect repair. Fontana G et al. used mineral-coated microparticles (MCMs) and fluoride MCM (FMCM) to deliver transforming growth factor 1 (TGFβ-1) cmRNA. After loading TGFβ-1 mRNA on FMCMs, they transfected bone marrow aspirate (BMAC) and then combined with autologous peripheral blood to form a clot, which was implanted into a 2.7 mm condyle osteochondral defect created in a rabbit knee. BMAC-TGFβ-1 mRNA regenerated cartilage, with increased type II collagen and glycosaminoglycan deposition, as well as reduced COL1 formation, but it did not significantly improve Odriscoll’s histology scores [57].

In our study, we found that injection of 2.5 µg and 5 µg cmBMP4 mRNA every 3 weeks for a total of three administrations prevented age-related cartilage loss and did not increase HO formation in the knee joints of aged mice. The increase in SOX9^+^ cells after IA injection of hBMP4 mRNA/LNP might contribute to the delayed cartilage degeneration. This contrasts with the need for a higher dose of cmhBMP4 mRNA for bone repair, likely because the knee joint contains cells that the injected mRNA can transfect and subsequently produce BMP4 protein to exert effects. Previous studies demonstrated that intra-articular injection of a lower dose of Runx1 mRNA (1 µg in 20 µL volume every 3 days for one month using PEG-PAsp(TET) rather than PEG-PAsp(DET)) promoted repair of OA induced by MCL and MM transection [51]. Using a novel articular cavity-localized lipid nanoparticle (LNP) named WG-PL14 to deliver a low dose of FGF18 mRNA by intra-articular injection (2 µg in 20 µL/knee once a week for 3 weeks) ameliorated anterior cruciate ligament transection (ACLT)-induced OA progression [55]. More recently, Kong K et al. reported that intra-articular injection of FGF18 mRNA/LNP (6 µg in 20 µL weekly) or FGF18 protein (500 ng in 20 µL weekly) for 8 weeks in the joint of DMM-induced and age-related OA models improved OA symptoms via activation of the FOX3A-autophage pathway, protecting chondrocytes from degeneration and senescence. It is noticeable that FGF18 protein administered at a much lower dose achieved a similar therapeutic effect to a much higher dose of FGF18 mRNA [56]. These studies, together with our study, indicate that smaller doses of cmRNA are required for cartilage repair and regeneration when compared to the higher dose needed for bone repair. Further, using BMPs to treat cartilage damage, especially osteochondral defects, may be more effective because BMPs not only promote cartilage repair but also promote subchondral bone defect healing, as we previously demonstrated using coacervate biomaterials to sustain the release of BMPs [9]. Therefore, cmRNA is more promising for cartilage repair.

## 5. Conclusions

In summary, we found that hBMP4 mRNA/LNP produced BMP4 protein in transfected hBMMSC cells and promoted osteogenic differentiation. Human BMP4 mRNA delivered with lipid nanoparticles promoted limited new bone formation and defect repair in critical-size calvarial bone defects in aged mice. By contrast, an intra-articular injection of a lower dose of BMP4 mRNA (ten times lower) improved cartilage repair and prevented cartilage loss in age-related OA. Therefore, hBMP4 mRNA may represent a novel therapy for the treatment of age-related OA.

## Data Availability

The raw data supporting the conclusions of this article will be made available by the authors on request.

## Abbreviations

BMP4	bone morphogenetic protein 4
Q-PCR	quantitative polymerase chain reaction
OA	osteoarthritis
MSCs	mesenchymal stem cells
TGFβ	transforming growth factor
mRNA	messenger ribonucleic acid
LNP	lipid nanoparticle
CT	computer tomography
OARSI	Osteoarthritis Research Society International
SOX9	Sry-box transcription factor 9
MIA	monosodium iodoacetate
COL1	collagen type 1
Ki67	marker of proliferation Kiel 67
PBS	phosphate-buffered saline
ELISA	Enzyme-Linked Immunosorbent Assay
COL2	collagen type 2
CTL	control
HO	heterotopic ossification
RUNX2	runt-related transcription factor 2
OSX	osterix
ALP	alkaline phosphatase
P16	P16ink4A
P21	P21CIP/Waf
FGF18	fibroblast growth factor 18
BV/TV	bone volume/total volume
Tb.Th	trabecular thickness
ACLT	anterior cruciate ligament transfection
